# Improved Survival with the Patients with Variceal Bleed

**DOI:** 10.4061/2011/356919

**Published:** 2011-07-07

**Authors:** Praveen Sharma, Shiv K. Sarin

**Affiliations:** Department of Hepatology and Transplant Hepatology, Institute of Liver & Biliary Sciences, New Delhi 110 070, India

## Abstract

Variceal hemorrhage is a major cause of death in patients with cirrhosis. Over the past two decades new treatment modalities have been introduced in the management of acute variceal bleeding (AVB) and several recent studies have suggested that the outcome of patients with cirrhosis and AVB has improved. Improved supportive measures, combination therapy which include early use of portal pressure reducing drugs with low rates of adverse effects (somatostatin, octerotide or terlipressin) and endoscopic variceal ligation has become the first line treatment in the management of AVB. Short-term antibiotic prophylaxis, early use of lactulose for prevention of hepatic encephalopathy, application of early transjugular intrahepatic portasystemic shunts (TIPS), fully covered self-expandable metallic stent in patients for AVB may be useful in those cases where balloon tamponade is considered. Early and wide availability of liver transplantation has changed the armamentarium of the clinician for patients with AVB. High hepatic venous pressure gradient (HVPG) >20 mmHg in AVB has become a useful predictor of outcomes and more aggressive therapies with early TIPS based on HVPG measurement may be the treatment of choice to reduce mortality further.

## 1. Introduction

Portal hypertension (PHT) worsens with increasing severity of cirrhosis and is responsible for many of its complications, which lead to clinical decompensation. The prevention and treatment of these complications have therefore been a cornerstone of the management of the patient with cirrhosis. Gastroesophageal varices are present in 50% of patients with cirrhosis, and variceal hemorrhage develops in up to one-third of these patients [1–3]. The initial appearance of varices in patients with compensated cirrhosis indicates a progression of the disease from a low-risk state to an intermediate one. Once bleeding occurs, this indicates decompensation and progression to a high risk of death [4, 5]. The risk of variceal hemorrhage is increased in patients who have large varices and advanced stages of liver disease, as assessed on the basis of the Child-Pugh class [6, 7]. Several studies published between 1942 and 1981 showed poor outcomes after variceal hemorrhage, with mortality rates of 40% at 6 weeks and 70% at 1 year [4, 8–11]. Over the past five decades, a number of randomized trials have shown an improvement in the efficacy of endoscopic, pharmacologic, surgical, and radiologic techniques for arresting hemorrhage [12–14]. Subsequently, retrospective single-center and multicenter studies have shown a decrease in hospital mortality associated with variceal hemorrhage over the past two decades [14–19]. 

In a study by Chalasani et al. [14] a total of 231 subjects were included, and their in-hospital, 6-week, and overall mortality rates were 14.2%, 17.5%, and 33.5%, respectively. The mortality rate after variceal bleeding in this study was substantially lower than previously reported. This suggests that advances made in the management of variceal bleeding have improved outcomes after variceal bleeding. Similarly Carbonell et al. [12] reviewed the clinical records of all patients with cirrhosis due to variceal bleeding during the years 1980, 1985, 1990, 1995, and 2000. Whereas balloon tamponade was still the first-line treatment in 1980, patients treated in 2000 received a vasoactive agent, an endoscopic treatment, and an antibiotic prophylaxis in, respectively, 90%, 100%, and 94% of cases. The in-hospital mortality rate steadily decreased over the study period: 42.6%, 29.9%, 25%, 16.2%, and 14.5% in 1980, 1985, 1990, 1995, and 2000, respectively (*P* < .05). Mortality decreased from 9% in 1980 to 0% in 2000 in Child-Turcotte-Pugh class A patients, from 46% to 0% in class B patients, and from 70% to 32% in class C patients. This improved survival was associated with a decrease of rebleeding (from 47% in 1980 to 13% in 2000) and bacterial infection rates (from 38% to 14%). On multivariable analysis, endoscopic therapy and antibiotic prophylaxis were independent predictors of survival. Thomopoulos et al. [18] studied 141 patients with acute variceal bleed and found 6-week, 1-year, and overall mortality were 12.1%, 18.4%, 32.6% and 48.2%, respectively. The rate of recurrent bleeding was 10.7% during initial hospitalisation. Being Child-Pugh C (*P* = .003) and shock on admission (*P* = .037) were independent predictors of 6-week mortality, while being Child-Pugh C (*P* = .028), presence of hepatocellular carcinoma or other neoplasia (*P* = .04), and partial thromboplastin time (*P* = .021) during the initial admission were independent predictors for 1-year mortality. Mortality was not affected by the presence of active bleeding and/or white nipple at emergency endoscopy. Also presence of infection was not an adverse factor of clinical outcome in our patients. In all these studies the decrease in mortality was largely due to improvement in general measures, more effective endoscopic therapy in combination with vasoactive medications, prevention of sepsis through the use of antibiotic prophylaxis, and the prevention of rebleeding.

## 2. Improvement in General Measures

There is evidence that current treatment strategies for acute variceal hemorrhage, including general and specific measures, have resulted in an improved survival [12, 18]. Initial resuscitation by multidisciplinary team involves basic measures including assessing the patient's airway and obtaining peripheral venous access. Blood volume resuscitation should be undertaken promptly but with caution, with the goals of maintaining hemodynamic stability and a hemoglobin of approximately 7-8 g/dL [19, 20]. This recommendation is based on experimental studies that show that restitution of all lost blood leads to increases in portal pressure to levels higher than baseline [21] and to more rebleeding and mortality [22]. Similarly, vigorous resuscitation with saline solution should generally be avoided because, in addition to possibly precipitating recurrent variceal hemorrhage, this can worsen or precipitate the accumulation of ascites or fluid at other extravascular sites.

## 3. Prophylactic Antibiotics in Acute Variceal Bleed

Currently, it is recommended that short-term antibiotic prophylaxis, a measure that reduces bacterial infections [23], variceal rebleeding, and death [24], be used in every patient with cirrhosis admitted with gastrointestinal hemorrhage [20, 25, 26]. Different antibiotics have been used in different trials compared with placebo (Table 1, [27–30]). Bacterial infection is commonly associated with variceal hemorrhage and appears to be an independent risk factor for failure to control bleeding [31] and predicts both early rebleeding and death [32, 33]. The routine use of prophylactic broad-spectrum antibiotics has shown a marked improvement in outcome in acute variceal hemorrhage. Routine intravenous ceftriaxone or postendoscopic norfloxacin reduces rebleeding rates compared to on-demand antibiotics (Table 2) [24, 29, 34–36]. A Cochrane meta-analysis of antibiotic prophylaxis in cirrhotic patients with gastrointestinal bleeding involving 12 trials with 1241 patients evaluated antibiotic prophylaxis compared with placebo or no antibiotic prophylaxis. Antibiotic prophylaxis compared with no intervention or placebo was associated with beneficial effects on mortality (RR 0.79, 95% CI 0.63 to 0.98), mortality from bacterial infections (RR 0.43, 95% CI 0.19 to 0.97), bacterial infections (RR 0.36, 95% CI 0.27 to 0.49). They concluded that prophylactic antibiotic use in patients with cirrhosis and upper gastrointestinal bleeding significantly reduced bacterial infections, and seems to have reduced all-cause mortality, bacterial infection mortality, rebleeding events, and hospitalisation length. These benefits were observed independently of the type of antibiotic used [37, 38]. The rationale behind the oral administration of norfloxacin, a poorly absorbed quinolone, is the selective eradication (or at least reduction) of Gram-negative bacteria in the gut, the source of bacteria. However, quinolone antibiotics with similar spectrum of activity, such as ciprofloxacin, could also be recommended. When oral administration is not possible, quinolones can be administered intravenously (IV). In a recent study performed in patients with advanced cirrhosis (Child B/C) and GI hemorrhage, IV ceftriaxone (1 g/day) was more effective than oral norfloxacin in preventing bacterial infections mostly those due to Gram-negative organisms [36]. It has now become standard practice to administer prophylactic antibiotics in acute variceal hemorrhage and in cirrhotic patients with gastrointestinal bleeding of any cause. The clear survival benefit associated with prophylactic antibiotics in gastrointestinal hemorrhage associated with cirrhosis is not in doubt. Both American and British guidelines recommend the administration of antibiotics prior to endoscopy in patients with AVB [39, 40].

## 4. Use of Newer Pharmacologic Treatment in Reducing Mortality

Pharmacological therapy has the advantages of being generally applicable and capable of being initiated as soon as a diagnosis of variceal hemorrhage is suspected, even prior to diagnostic EGD [20, 25, 26]. Newer development of drugs like somatostatin and analogues such as octreotide and vapreotide also causes splanchnic vasoconstriction at pharmacological doses due to an inhibition of the release of vasodilatory peptides mainly glucagon. The advantage of somatostatin and analogues such as octreotide and vapreotide is that they are safe and can be used continuously for 5 days or even longer [20]. 

However, results of meta-analysis of trials of octreotide are controversial [41, 42]. In a recent metaanalysis twenty studies were identified for all the comparison groups that indicates that terlipressin was associated with a statistically significant reduction in all-cause mortality compared to placebo (relative risk 0.66, 95% confidence interval 0.49 to 0.88). There was no significant difference between the terlipressin group and any of the comparison groups in the number of adverse events that caused death or withdrawal of medication. On the basis of a 34% relative risk reduction in mortality, terlipressin should be considered to be effective in the treatment of acute variceal hemorrhage [43].

Endoscopic therapy with either band ligation or injection sclerotherapy is an integral component of the management of acute variceal bleeding and of the long-term treatment of patients after a variceal bleed. Regarding the best endoscopic therapy, a metaanalysis of 10 randomized controlled trials including 404 patients shows an almost significant benefit of EVL in the initial control of bleeding compared to sclerotherapy (pooled relative risk of 0.53 with a confidence interval of 0.28–1.01) [44]. Variceal eradication with endoscopic ligation requires fewer endoscopic treatment sessions and causes substantially less esophageal complications than does injection sclerotherapy. Although the incidence of early gastrointestinal rebleeding is reduced by endoscopic ligation in most studies, there is no overall survival benefit relative to injection sclerotherapy.

In a recent metaanalysis pharmacotherapy is found to be as effective as emergency sclerotherapy in patients with acute variceal bleed. Seventeen trials including 1817 patients were identified. No significant differences were found comparing sclerotherapy with each vasoactive drug for any outcome. Combining all the trials irrespective of the vasoactive drug, the risk differences (95% confidence intervals) were failure to control bleeding −0.02 (−0.06 to 0.02), five-day failure rate −0.05 (−0.10 to 0.01), rebleeding 0.01 (−0.03 to 0.05), mortality (17 randomised trials, 1817 patients) −0.02 (−0.06 to 0.02), and transfused blood units (8 randomised trials, 849 patients) (weighted mean difference) −0.24 (−0.54 to 0.07). Adverse events 0.08 (0.03 to 0.14) and serious adverse events 0.05 (0.02 to 0.08) were significantly more frequent with sclerotherapy [45].

## 5. Combination Therapy as Standard of Therapy

Combination of both pharmacologic and endoscopic therapy in the treatment of AVB is strongly supported by numerous trials showing that the efficacy of both emergency EST and EBL is significantly improved when they are associated with pharmacologic treatment [41, 46]. Although both methods are highly effective in controlling AVB, EBL has become the treatment of choice both for controlling variceal hemorrhage and for variceal obliteration in secondary prophylaxis [20, 26]. A meta-analysis has shown that EBL is better than EST for all major outcomes including initial control of bleeding, recurrent bleeding, side effects, time to variceal obliteration, and survival [47]. Thus, combination therapy with a vasoactive drug plus EBL is considered the standard of care for AVB, and it is currently recommended by guidelines [20]. Combination therapy improves the 5-day success rate compared with endoscopic ligation therapy alone [48, 49], but this is not associated with any differences in mortality. Given these reasons, EBL at present is the endoscopic method of choice to treat esophageal varices in most cases. However, EST is an accepted method if EBL cannot be performed.

## 6. Evaluation of Hepatic Venous Pressure Measurement in Patient with Acute Variceal Bleed

Assessment of portal pressure by the hepatic venous pressure gradient (HVPG) has been a useful predictor of outcomes in both stages. In patients with compensated cirrhosis, an HVPG greater or equal to 10 mmHg is the most important predictor of the development of varices and clinical decompensation [50, 51]. Prospective cohort studies in which HVPG has been measured within 48 hours of admission for hemorrhage show that levels greater than 20 mmHg are associated with increased rebleeding and mortality [52–54].

A more recent study performed in the era of combined vasoactive drug plus endoscopic therapy confirms this HVPG cutoff and shows that an index including CTP score and blood pressure at admission has similar prognostic value [55]. Furthermore, a drug-induced HVPG reduction of less than 10% predicts 5-day failure. This response may improve by doubling the dose of somatostatin or switching to another agent (such as terlipressin).

In acute complications of cirrhosis, such as variceal bleeding, there have been fewer studies of portal pressure, but, also in this setting, HVPG has been shown to be prognostic for both survival and the course of bleeding. Vinel et al. [56] documented that short-term prognosis in alcoholic cirrhotic patients with variceal bleeding was independently associated with portohepatic gradient measured within 48 h of admission. This was confirmed in a small study of 22 patients, in which the best cutoff for continued bleeding or early rebleeding was HVPG >16 mmHg [53]. Villanueva et al. [57] showed that HVPG > 20 mmHg and a decrease <10 mmHg under vasoactive therapy were independent predictors of further bleeding. An HVPG > 20 mmHg has been shown to correlate with important clinical outcomes such as more difficulty in controlling acute variceal bleeding, more early rebleeding, more blood transfusion need, more days in intensive care and increased hospital mortality [58]. Lastly Avgerinos et al. [59] showed that HVPG > 16 mmHg was independently associated with death and/or early rebleeding evaluating HVPG measurements before and immediately after endoscopic treatment and every 24 h for a 5-day period.

## 7. Transjugular Intrahepatic Portosystemic Shunt in Acute Variceal Hemorrhage

Transjugular intrahepatic portosystemic shunt (TIPS) is a reasonable alternative in the face of failure of combined pharmacologic plus endoscopic therapy. In the Baveno conference, it was considered that a second attempt at endoscopic therapy was one possibility but that one could perform TIPS after failure of the first endoscopic therapy. An elevated hepatic venous pressure gradient (>20 mm Hg) measured within 24 hours after the start of bleeding is the best predictor of treatment failure [26]. The use of TIPS to control variceal bleeding has largely been reserved for patients who require rescue therapy because hemostasis has not been achieved, either during the index bleeding or during the secondary-prophylaxis period. TIPS is extremely effective in controlling bleeding, with a reported rate of immediate hemostasis of 93% and with rebleeding in only 12% of patients. Nevertheless, mortality at 6 weeks among patients treated with rescue TIPS for uncontrolled index bleeding and rebleeding is very high (35%), reflecting the severity of their underlying liver disease as well as additional organ dysfunction that may have occurred owing to hypotension, infection, and aspiration [60].

Recently García-Pagán and colleagues report the results of a randomized, multicenter study that compared early TIPS with optimal medical therapy (endoscopic therapy plus vasoactive drugs) in patients at high risk for rebleeding who were either in Child-Pugh class B with active bleeding at endoscopy or in Child-Pugh class C. This study shows the benefit of early TIPS in patients with Child-Pugh class B or C disease who-are at high risk for uncontrolled bleeding with standard therapy. Patients who were randomly assigned to receive TIPS had a significantly better chance of remaining free of bleeding than did those who received the standard care (97% versus 50%), possibly owing to a greater reduction in portal pressure with TIPS than could be achieved with pharmacologic therapy. The rate of survival at 6 weeks was 97% in the TIPS group as compared with 67% in the medical therapy group, as a result of reductions in rebleeding, sepsis and liver failure [61]. Use of the newer stents, which are covered with extended polytetrafluoroethylene (e-PTFE), probably has an important bearing on the outcome of this study [15].

## 8. Newer Methods

The recent introduction of a fully covered self-expandable metallic stent for AVB may be useful in those cases where balloon tamponade is considered. The stent is placed over a guide wire previously passed to the stomach. The stent has a distal balloon that is inflated with a syringe to ensure proper location in the cardias and lower esophagus so no fluoroscopy is needed. The stent can be left in place for up to 14 days, and it can be retrieved by endoscopy with a hook system. There are limited data with its use. A pilot study of 20 patients who failed standard of care treatment reported 100% success without any significant complications [62].

## 9. Summary

AVB is a dreaded complication of patients with portal hypertension. Initial management includes appropriate volume replacement, transfusion of blood to keep hemoglobin levels at 7-8 g/L, antibiotic prophylaxis, and endotracheal intubation in selected cases. Standard of care mandates for early administration of vasoactive drug therapy and then EBL or injection ES (if EBL cannot be performed) within the first 12 hours of the index bleed. The use of pharmacologic agents may be prolonged for up to 5 days. Patients who fail endoscopic therapy may require temporary placement of balloon tamponade or stents. All patients surviving an episode of AVB should undergo further prophylaxis to prevent rebleeding. However, despite the application of these gold-standard treatments, 10% to 15% of cirrhotic patients still have treatment failure. Despite the high success of rescue TIPS in controlling bleeding in treatment failures, the mortality of patients in whom the initial approach failed is high due to liver failure. It is possible that in the near future, patients may be treated ‘‘á la carte.” Indeed, in high-risk patients, more aggressive therapies with early PTFE TIPS based on HVPG measurement may be the treatment of choice to reduce mortality further.

## Figures and Tables

**Table 1:** Antibiotics compared to placebo in acute variceal bleed.

Author	Outcome	Drugs	Placebo (*n*) *P*	Antibiotics (*n*) *A*	Infections *P* versus *A*	efficacy
Pauwels et al. [27]	Bacterial infections	ciprofloxacin and a amoxicillin and clavulanic acid	34	30	53% versus 13%	*A* > *P*
Soriano et al. [30]	Bacterial infection	Norflox	59	60	10% versus 37%	*A* > *P*
Hsieh et al. [28]	Bacterial infection	ciprofloxacin	60	60	45% versus 10%	*A* > *P*
Jun et al. [29]	Bacterial infection	Cefotaxime	62	58	16% versus 3%	*A* > *P*

**Table 2:** Antibiotics preventing mortality in acute variceal bleed.

Author	Outcome	Drug	Drug	Relative risk	CI
Gulberg et al. [34]	Bacterial infection	Ceftriaxone 1 gm (1/40)	Ceftriaxone 2 gm (1/42)	1.05	0.11–9.80
Lata et al. [35]	Mortality	Ampicillin and sulbactam 3 g (12/21)	Norfloxacin 800 mg (7/25)	2.04	0.98–4.23
Fernández et al. [36]	Mortality	Ceftriaxone 1 g (8/54)	Norfloxacin 800 mg (6/57)	1.41	0.52–3.79

## References

[1] de Franchis R, Primignani M (2001). Natural history of portal hypertension in patients with cirrhosis. *Clinics in Liver Disease*.

[2] Sanyal AJ, Fontana RJ, DiBisceglie AM (2006). The prevalence and risk factors associated with esophageal varices in subjects with hepatitis C and advanced fibrosis. *Gastrointestinal Endoscopy*.

[3] D’Amico G, Garcia-Tsao G, Pagliaro L (2006). Natural history and prognostic indicators of survival in cirrhosis. A systematic review of 118 studies. *Journal of Hepatology*.

[4] Graham DY, Smith JL (1981). The course of patients after variceal hemorrhage. *Gastroenterology*.

[5] Smith JL, Graham DY (1982). Variceal hemorrhage: a critical evaluation of survival analysis. *Gastroenterology*.

[6] The North Italian Endoscopic Club for the Study and Treatment of Esophageal Varices (1988). Prediction of the first variceal hemorrhage in patients with cirrhosis of the liver and esophageal varices. A prospective multicenter study. *The New England Journal of Medicine*.

[7] D’Amico G, Morabito A, Pagliaro L, Marubini E (1986). Survival and prognostic indicators in compensated and decompensated cirrhosis. *Digestive Diseases and Sciences*.

[8] Nachlas MM, O'Neil JE, Campbell AJ (1955). The life history of patients with cirrhosis of the liver and bleeding esophageal varices. *Annals of Surgery*.

[9] Powell WJ, Klatskin G (1968). Duration of survival in patients with Laennec’s cirrhosis: influence of alcohol withdrawal, and possible effects of recent changes in general management of the disease. *The American Journal of Medicine*.

[10] Pinto HC, Abrantes A, Esteves AV, Almeida H, Correia JP (1989). Long-term prognosis of patients with cirrhosis of the liver and upper gastrointestinal bleeding. *The American Journal of Gastroenterology*.

[11] Garceau AJ, Chalmers TC (1963). The natural history of cirrhosis. I. Survival with esophageal varices. *The New England Journal of Medicine*.

[12] Carbonell N, Pauwels A, Serfaty L, Fourdan O, Lévy VG, Poupon R (2004). Improved survival after variceal bleeding in patients with cirrhosis over the past two decades. *Hepatology*.

[13] El-Serag HB, Everhart JE (2000). Improved survival after variceal hemorrhage over an 11-year period in the Department of Veterans Affairs. *The American Journal of Gastroenterology*.

[14] Chalasani N, Kahi C, Francois F (2003). Improved patient survival after acute variceal bleeding: a multicenter, cohort study. *The American Journal of Gastroenterology*.

[15] Angermayr B, Cejna M, Koenig F (2003). Survival in patients undergoing transjugular intrahepatic portosystemic shunt: ePTFE-covered stent grafts versus bare stents. *Hepatology*.

[16] McCormick PA, O’Keefe C (2001). Improving prognosis following a first variceal haemorrhage over four decades. *Gut*.

[17] D'Amico G, de Franchis R, Cooperative Study Group (2003). Upper digestive bleeding in cirrhosis. Post-therapeutic outcome and prognostic indicators. *Hepatology*.

[18] Thomopoulos K, Theocharis G, Mimidis K, Lampropoulou-Karatza C, Alexandridis E, Nikolopoulou V (2006). Improved survival of patients presenting with acute variceal bleeding. Prognostic indicators of short- and long-term mortality. *Digestive and Liver Disease*.

[19] de Franchis R (2005). Evolving consensus in portal hypertension. Report of the Baveno IV consensus workshop on methodology of diagnosis and therapy in portal hypertension. *Journal of Hepatology*.

[20] de Franchis R (2010). Revising consensus in portal hypertension: report of the Baveno V consensus workshop on methodology of diagnosis and therapy in portal hypertension. *Journal of Hepatology*.

[21] Kravetz D, Sikuler E, Groszmann RJ (1986). Splanchnic and systemic hemodynamics in portal hypertensive rats during hemorrhage and blood volume restitution. *Gastroenterology*.

[22] Castañeda B, Morales J, Lionetti R (2001). Effects of blood volume restitution following a portal hypertensive-related bleeding in anesthetized cirrhotic rats. *Hepatology*.

[23] Bernard B, Grange JD, Khac EN, Amiot X, Opolon P, Poynard T (1999). Antibiotic prophylaxis for the prevention of bacterial infections in cirrhotic patients with gastrointestinal bleeding: a meta-analysis. *Hepatology*.

[24] Hou MC, Lin HC, Liu TT (2004). Antibiotic prophylaxis after endoscopic therapy prevents rebleeding in acute variceal hemorrhage: a randomized trial. *Hepatology*.

[25] Garcia-Tsao G, Sanyal AJ, Grace ND (2007). Prevention and management of gastroesophageal varices and variceal hemorrhage in cirrhosis. *The American Journal of Gastroenterology*.

[26] Garcia-Tsao G, Sanyal AJ, Grace ND, Carey W (2007). Prevention and management of gastroesophageal varices and variceal hemorrhage in cirrhosis. *Hepatology*.

[27] Pauwels A, Mostefa-Kara N, Debenes B, Degoutte E, Lévy VG (1996). Systemic antibiotic prophylaxis after gastrointestinal hemorrhage in cirrhotic patients with a high risk of infection. *Hepatology*.

[28] Hsieh WJ, Lin HC, Hwang SJ, Hou MC, Lee FY (1998). The effect of ciprofloxacin in the prevention of bacterial infection in patients with cirrhosis after upper gastrointestinal bleeding. *The American Journal of Gastroenterology*.

[29] Jun CH, Park CH, Lee WS, Joo YE, Kim HS, Choi SK (2006). Antibiotic prophylaxis using third generation cephalosporins can reduce the risk of early rebleeding in the first acute gastroesophageal variceal hemorrhage: a prospective randomized study. *Journal of Korean Medical Science*.

[30] Soriano G, Guarner C, Tomás A (1992). Norfloxacin prevents bacterial infection in cirrhotics with gastrointestinal hemorrhage. *Gastroenterology*.

[31] Goulis J, Armonis A, Patch D, Sabin C, Greenslade L, Burroughs AK (1998). Bacterial infection is independently associated with failure to control bleeding in cirrhotic patients with gastrointestinal hemorrhage. *Hepatology*.

[32] Bernard B, Cadranel JF, Valla D, Escolano S, Jarlier V, Opolon P (1995). Prognostic significance of bacterial infection in bleeding cirrhotic patients: a prospective study. *Gastroenterology*.

[33] Garden OJ, Motyl H, Gilmour WH, Utley RJ, Carter DC (1985). Prediction of outcome following acute variceal haemorrhage. *The British Journal of Surgery*.

[34] Gulberg V, Deibert P, Ochs A, Rossle M, Gerbes AL (1999). Prevention of infectious complications after transjugular intrahepatic portosystemic shunt in cirrhotic patients with a single dose of ceftriaxone. *Hepato-Gastroenterology*.

[35] Lata J, Juránková J, Husová L (2005). Variceal bleeding in portal hypertension: bacterial infection and comparison of efficacy of intravenous and per-oral application of antibiotics—a randomized trial. *The European Journal of Gastroenterology and Hepatology*.

[36] Fernández J, del Arbol LR, Gómez C (2006). Norfloxacin vs ceftriaxone in the prophylaxis of infections in patients with advanced cirrhosis and hemorrhage. *Gastroenterology*.

[37] Soares-Weiser K, Brezis M, Tur-Kaspa R, Leibovici L (2002). Antibiotic prophylaxis for cirrhotic patients with gastrointestinal bleeding. *Cochrane Database of Systematic Reviews*.

[38] Chavez-Tapia NC, Barrientos-Gutierrez T, Tellez-Avila FI, Soares-Weiser K, Uribe M (2010). Antibiotic prophylaxis for cirrhotic patients with upper gastrointestinal bleeding. *Cochrane Database of Systematic Reviews*.

[39] Allison MC, Sandoe JA, Tighe R, Simpson IA, Hall RJ, Elliott TS (2009). Antibiotic prophylaxis in gastrointestinal endoscopy. *Gut*.

[40] Banerjee S, Shen B, Baron TH (2008). Antibiotic prophylaxis for GI endoscopy. *Gastrointestinal Endoscopy*.

[41] D’Amico G, Pagliaro L, Bosch J (1999). Pharmacological treatment of portal hypertension: an evidence-based approach. *Seminars in Liver Disease*.

[42] Corley DA, Cello JP, Adkisson W, Ko W-F, Kerlikowske K (2001). Octreotide for acute esophageal variceal bleeding: a meta-analysis. *Gastroenterology*.

[43] Ioannou G, Doust J, Rockey DC (2003). Terlipressin for acute esophageal variceal hemorrhage. *Cochrane Database of Systematic Reviews*.

[44] Jutabha R, Jensen DM, Martin P, Savides T, Han SH, Gornbein J (2005). Randomized study comparing banding and propranolol to prevent initial variceal hemorrhage in cirrhotics with high-risk esophageal varices. *Gastroenterology*.

[45] D’Amico G, Pagliaro L, Pietrosi G, Tarantino I (2010). Emergency sclerotherapy versus vasoactive drugs for bleeding oesophageal varices in cirrhotic patients. *Cochrane Database of Systematic Reviews*.

[46] Qureshi W, Adler DG, Davila R (2005). ASGE guideline: the role of endoscopy in the management of variceal hemorrhage, updated. *Gastrointestinal Endoscopy*.

[47] Laine L, El-Newihi HM, Migikovsky B, Sloane R, Garcia F (1993). Endoscopic ligation compared with sclerotherapy for the treatment of bleeding esophageal varices. *Annals of Internal Medicine*.

[48] Villanueva C, Ortiz J, Sàbat M (1999). Somatostatin alone or combined with emergency sclerotherapy in the treatment of acute esophageal variceal bleeding: a prospective randomized trial. *Hepatology*.

[49] Baares R, Albillos A, Rincón D (2002). Endoscopic treatment versus endoscopic plus pharmacologic treatment for acute variceal bleeding: a meta-analysis. *Hepatology*.

[50] Groszmann RJ, Garcia-Tsao G, Bosch J (2005). Beta-blockers to prevent gastroesophageal varices in patients with cirrhosis. *The New England Journal of Medicine*.

[51] Ripoll C, Groszmann R, Garcia-Tsao G (2007). Hepatic venous pressure gradient predicts clinical decompensation in patients with compensated cirrhosis. *Gastroenterology*.

[52] Moitinho E, Escorsell A, Bandi JC (1999). Prognostic value of early measurements of portal pressure in acute variceal bleeding. *Gastroenterology*.

[53] Ready JB, Robertson AD, Goff JS, Rector WG (1991). Assessment of the risk of bleeding from esophageal varices by continuous monitoring of portal pressure. *Gastroenterology*.

[54] Villanueva C, Ortiz J, Minana J (2001). Somatostatin treatment and risk stratification by continuous portal pressure monitoring during active variceal bleeding. *Gastroenterology*.

[55] Abraldes JG, Villanueva C, Banares R (2008). Hepatic venous pressure gradient and prognosis in patients with acute variceal bleeding treated with pharmacologic and endoscopic therapy. *Journal of Hepatology*.

[56] Vinel JP, Cassigneul J, Levade M, Voigt JJ, Pascal JP (1986). Assessment of short-term prognosis after variceal bleeding in patients with alcoholic cirrhosis by early measurement of portohepatic gradient. *Hepatology*.

[57] Villanueva C, Ortiz J, Miana J (2001). Somatostatin treatment and risk stratification by continuous portal pressure monitoring during acute variceal bleeding. *Gastroenterology*.

[58] Monescillo A, Martínez-Lagares F, Ruiz-Del-Arbol L (2004). Influence of portal hypertension and its early decompression by TIPS placement on the outcome of variceal bleeding. *Hepatology*.

[59] Avgerinos A, Armonis A, Stefanidis G (2004). Sustained rise of portal pressure after sclerotherapy, but not band ligation, in acute variceal bleeding in cirrhosis. *Hepatology*.

[60] Boyer TD, Haskal ZJ (2005). The role of transjugular intrahepatic portosystemic shunt in the management of portal hypertension. *Hepatology*.

[61] García-Pagán JC, Caca K, Bureau C (2010). Early use of TIPS in patients with cirrhosis and variceal bleeding. *The New England Journal of Medicine*.

[62] Hubmann R, Bodlaj G, Czompo M (2006). The use of self-expanding metal stents to treat acute variceal bleeding. *Endoscopy*.

